# Sexual Dimorphic Regulation of Body Weight Dynamics and Adipose Tissue Lipolysis

**DOI:** 10.1371/journal.pone.0037794

**Published:** 2012-05-25

**Authors:** Verena Benz, Mandy Bloch, Sami Wardat, Christian Böhm, Lukas Maurer, Shokoufeh Mahmoodzadeh, Petra Wiedmer, Joachim Spranger, Anna Foryst-Ludwig, Ulrich Kintscher

**Affiliations:** 1 Center for Cardiovascular Research, Institute of Pharmacology, Charité-Universitätsmedizin Berlin, Berlin, Germany; 2 Department of Endocrinology, Diabetes, and Nutrition, Charité-Universitätsmedizin Berlin, Berlin, Germany; 3 Center for Cardiovascular Research, Institute of Gender in Medicine, Charité-Universitätsmedizin Berlin, Berlin, Germany; 4 Genetics of Metabolic and Reproductive Disorders, Max-Delbrueck-Center for Molecular Medicine, Berlin, Germany; Karolinska Insitutet, Sweden

## Abstract

**Background:**

Successful reduction of body weight (BW) is often followed by recidivism to obesity. BW-changes including BW-loss and -regain is associated with marked alterations in energy expenditure (EE) and adipose tissue (AT) metabolism. Since these processes are sex-specifically controlled, we investigated sexual dimorphisms in metabolic processes during BW-dynamics (gain-loss-regain).

**Research Design:**

Obesity was induced in C57BL/6J male (m) and female (f) mice by 15 weeks high-fat diet (HFD) feeding. Subsequently BW was reduced (-20%) by caloric restriction (CR) followed by adaptive feeding, and a regain-phase. Measurement of EE, body composition, blood/organ sampling were performed after each feeding period. Lipolysis was analyzed ex-vivo in gonadal AT.

**Results:**

Male mice exhibited accelerated BW-gain compared to females (relative BW-gain m:140.5±3.2%; f:103.7±6.5%; p<0.001). In consonance, lean mass-specific EE was significantly higher in females compared to males during BW-gain. Under CR female mice reached their target-BW significantly faster than male mice (m:12.2 days; f:7.6 days; p<0.001) accompanied by a sustained sex-difference in EE. In addition, female mice predominantly downsized gonadal AT whereas the relation between gonadal and total body fat was not altered in males. Accordingly, only females exhibited an increased rate of forskolin-stimulated lipolysis in AT associated with significantly higher glycerol concentrations, lower RER-values, and increased AT expression of adipose triglyceride lipase (ATGL) and hormone sensitive lipase (HSL). Analysis of AT lipolysis in estrogen receptor alpha (ERα)–deficient mice revealed a reduced lipolytic rate in the absence of ERα exclusively in females. Finally, re-feeding caused BW-regain faster in males than in females.

**Conclusion:**

The present study shows sex-specific dynamics during BW-gain-loss-regain. Female mice responded to CR with an increase in lipolytic activity, and augmented lipid-oxidation leading to more efficient weight loss. These processes likely involve ERα-dependent signaling in AT and sexual dimorphic regulation of genes involved in lipid metabolism.

## Introduction

Worldwide the occurrence of obesity is dramatically increasing in the last decades. BW reduction ameliorates all aspects of metabolic alterations, but the benefit appears to persist only as long as weight reduction is maintained. However, most therapeutic approaches do not induce a sustained weight loss and obese dieters fail to maintain their reduced BW. The metabolic defense against the reduction of BW is reflected in a decreased EE [1], [2]. These compensatory changes in EE oppose the maintenance of reduced BW and likely cause the recidivism to obesity. As shown in multiple studies, these processes are mediated by a coordinated endocrine response involving leptin, ghrelin, thyroxin, and other hormones modifying food intake, EE and factors that influence BW-regulation [3]–[5]. In addition, there is a growing body of evidence from human and rodent studies for a crucial role of estrogens in the regulation of BW-loss, -maintenance, and -regain. As shown previously the systemic loss of ligand-mediated estrogen receptor (ER)-signaling at menopause is associated with increased adiposity, in particular in visceral fat depots and body fat redistribution [6]. The accumulation of visceral fat depots increases the risk for the development of type 2 diabetes and other diseases associated with the metabolic syndrome [7]. The restoration of regular ER-signaling by hormone replacement can prevent menopause-induced gains in fat mass, and results in fat redistribution to subcutaneous fat depots, and improvement of insulin sensitivity [8]. These studies are further supported by preclinical studies in female rodents who became obese after undergoing ovariectomy. Replacement of estrogens in these ovariectomized animals abrogated the BW-gain [9]. Similar to the ligand-deficient models the ERα-knock out mice exhibit increased BW and AT-mass without a concomitant change in food consumption [10]. EE was significantly reduced in ERα-deficient mice compared to wild-type animals suggesting an augmented substrate utilization and metabolism by ERα activation, underlining the role of ERα in EE [10], [11]. In addition to ERα-regulated EE, other authors have favored direct actions of ERα in adipocytes including a prominent inhibition of lipogenesis [12] or direct activation of lipolysis (reviewed in [13]). Thus, actions of ERα in AT may play an important role in mediating the effects of receptor-dependent estrogen actions on BW-regulation. We could previously show that also ERβ-deficient mice develop higher BW and AT-mass under HFD associated with marked increase of food efficiency (ratio of BW-gain and food intake) and attenuated fatty acid oxidation [14]. Taken together, these data suggest an involvement of estrogenic signaling in the regulation of BW and AT-metabolism likely resulting in sexual dimorphisms during BW-gain, -loss, and -regain.

During CR a negative energy balance causes the breakdown of triglycerides in adipocytes, and the AT provides free fatty acids (FFA) to the skeletal muscle and peripheral tissues as energy source. Lipolysis is controlled by a multi-enzyme complex of co-regulators and -activators and is mostly mediated by the activation of two main lipases ATGL and HSL [15].

Studies focussing on sex-specific differences demonstrated consistent results with regard to lipolysis and fat oxidation during exercise. When compared with men, women exhibited an improved ultra-endurance capacity, and higher rates of fat oxidation [16]. Along this line, skeletal muscle from women show higher mRNA expression of genes involved in lipid metabolism such as fatty acid transporters and enzymes involved in ß-oxidation, when compared to men [17], [18]. In consonance, our group could recently demonstrate that female mice have a significantly higher exercised-induced lipolytic rate compared to male mice undergoing forced treadmill-training [19]. Thus, we hypothesized that also under other conditions inducing a negative energy balance such as CR during weight reduction sex-specific differences in lipolysis may occur.

To investigate further sex-specific differences in BW-regulation and lipolysis we established a model of BW-gain, -loss, and -regain in which female and male mice were fed a HFD to induce obesity (DIO =  diet-induced obesity). Subsequently BW was reduced and maintained by adaptive feeding. Regain of reduced BW was achieved by ad libitum re-feeding. The major focus of the present study was on the BW-gain and –loss phase. This model may provide a useful tool to identify sex-specific differences in the dynamics of BW-gain, -loss and –regain.

## Materials and Methods

### Ethics Statement

All animal procedures were performed in accordance with the guidelines of the Charité Medical University Berlin and this study was specifically approved by the Landesamt für Gesundheit und Soziales (LaGeSo, Berlin, Germany) for the use of laboratory animals and followed the current version of the German Law on the Protection of Animals.

### Animals and the Model of BW- changes

**Figure 1:**
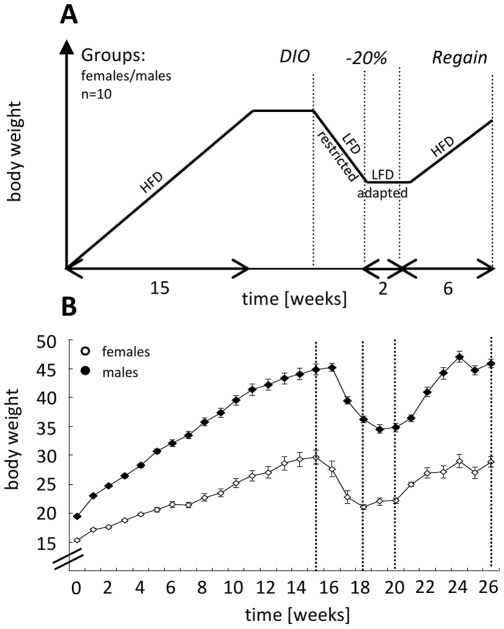
Animal model. A: Scheme of the feeding protocol to induce body weight changes (DIO =  diet-induced obesity). **B**: Original BW data of female/male mice throughout the feeding protocol.

Five-week-old female and male C57BL/6J mice were housed in a temperature controlled facility (25°C) with a 12 h light-dark cycle. Mice were fed ad libitum with a HFD (Research Diets, 60% kcal from fat, 20% kcal from carbohydrates, 20% kcal from protein) for 15 weeks to induce obesity. To assure the investigation of age-matched groups the initial HFD feeding period was defined as a stable parameter in our protocol (15 weeks). BW and food consumption were monitored twice a week. After 15 weeks mice were randomized into three groups: a diet-induced obesity group (DIO), a group that underwent caloric restriction (CR) until reaching a defined target body weight (−20% of DIO-BW), and a group that underwent CR with subsequent regain by ad libitum re-feeding for 6 weeks (Fig. 1) (n = 10 males and n = 10 females in each group). Animals undergoing CR were single caged from week 15, and after one week of adaption food intake was daily quantified including calculation of caloric intake. CR started after one week, and caloric intake per day was reduced by 50% and changed to a low-fat diet (LFD, Research Diets 10% kcal from fat, 70% kcal from carbohydrates, 20% kcal from protein). Mice were fed daily at 5 p.m., and BW was monitored before feeding. After reaching the target BW of −20%, weight was maintained by adaptive feeding for 2 weeks. After this period mice were re-fed ad libitum with HFD to cause a rebound of BW. Mice were sacrificed in the DIO phase (DIO group), after reaching the target BW (−20% group) and after regaining BW (regain group) (Fig. 1). Blood samples were collected when sacrificing the animals (DIO, −20%) and during weight loss (day 3 of CR) by retroorbital venous puncture under short isoflurane anesthesia, and serum was frozen at −20°C. Organs were frozen in liquid nitrogen, and gonadal fat pads were partly taken for an ex-vivo lipolysis assay. Gonadal fat pad mass was quantified before freezing. Tissue from ERα–deficient mice was provided by S. Mahmoodzadeh.

### Metabolic Cage System

Physical activity and EE were monitored using a combined indirect calorimetry system (TSE Systems GmbH, Bad Homburg, Germany). After adaptation, physical activity was determined for 23 h using a multi-dimensional infrared light beam system. The respiratory exchange ratio (RER) was calculated as the ratio between CO_2_ produced (V_CO2_) and O_2_ consumed (V_O2_). The EE was normalized to the lean body mass to avoid the possible confounding effects from diverged BW and lean mass. Since weekly BW gain under HFD decreases by time of feeding, 4 weeks HFD were chosen as a period of maximal BW gain for the first analysis under HFD. This phenotyping was accompanied by additional measurements after 15 weeks (DIO) and during CR.

### NMR

Body composition was determined by nuclear magnetic resonance imaging (Echo MRI mouse, Echo Medical Systems, Houston, USA) before metabolic phenotyping and sacrificing the animals.

### Serum Glycerol

For the determination of free serum glycerol a free glycerol kit was used (Sigma), and the procedure followed the instruction of the manufacturer.

### Liver Triglycerides

100 mg liver tissue was homogenized in 1 ml of chloroform-methanol-water-mix (3∶1.5∶1) with a tissue ruptor followed by sonification. After addition of 1 ml H_2_O and 2 ml chloroform samples were centrifuged. The supernatant was evaporated at 70°C, and triglycerides were resuspended in isopropanol. The concentration of triglycerides was determined with a Triglycerides FS Kit (Diasys, Holzheim, Germany) according to the manufacturer’s instructions.

### Ex-vivo Lipolysis Assay in Gonadal AT- explants

Gonadal fat pads were surgically removed from female and male mice (n = 6−7), and washed with ice-cold PBS. AT excised pads (∼ 3 mm×3 mm, n = 4/mouse) were preincubated for 2 h in 140 µl DMEM (Life Technologies) containing 2% fatty acid free serum albumin (Sigma-Aldrich). Subsequently fat pads were incubated in 140 µl DMEM +2% BSA (fatty acid free) in the presence of forskolin (10 µM) for 1 h at 37°C. Afterwards, FFA content was quantified for each sample in the medium using the HR-NEFA series (Wako Diagnostics). For protein determination, the fat pads were washed in PBS, and triglycerides were extracted using chloroform/methanol (2∶1) for ∼1 h at 45°C under vigorous shaking. Thereafter, fat free pads were lysed in 0.3 N NaOH/0.1% SDS overnight at 55°C under vigorous shaking. Next, protein concentration was measured using the BCA reagent (Pierce). FFA release from each sample was normalized to protein content of each fat pad used in the experiment. For statistical analysis the mean for each mouse was calculated.

### RNA Analysis

Total RNA from gonadal AT was isolated using Qiazol and RNeasy Micro kit (Qiagen), according to the manufacturer’s instruction. For real-time PCR analysis, RNA samples were DNAse digested (Qiagen); reverse transcribed using reverse transcriptase, RNAsin, and dNTPs (Promega), according to the manufacturer’s instructions; and used in quantitative PCR (qPCR) reactions in the presence of a fluorescent dye (Sybrgreen, Life Sciences). Relative abundance of mRNA was calculated after normalization to 18 S ribosomal. The primer sequences used for the measurements are available on demand.

### Statistical Analysis

Comparison of mean values between groups was evaluated by two-way ANOVA (Bonferroni posttest), two-way ANOVA with repeated measures (Bonferroni posttest), or unpaired *t*-tests, as appropriate, analyzed with GraphPad Prism Software. Statistical significance was assumed at p<0.05. Vertical lines in the histograms indicate means ±SEM. The n-number is indicated for each experiment.

## Results

As described in detail in the method section, a model of BW-changes including a gain phase, a reduction phase, a maintenance phase, and a regain phase was established (Figure 1A+B).

### Weight Gain Phase

**Figure 2:**
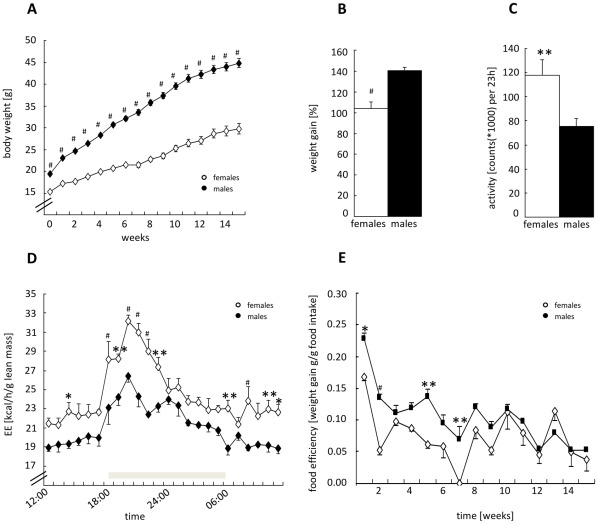
Weight gain phase. A: Body weight development of female/male mice showed a sex-specific difference in 15 weeks of HFD-feeding. Shown are the means ±SEM of body weight, measured weekly [n = 20 mice/group, two-way ANOVA with repeated measures] (factor interaction: p_sex/time_<0.001). **B**: Relative weight gain in female/male mice after 15 weeks of HFD feeding expressed as percent change from initial weight [n = 10 mice/group, unpaired *t*-test]. **C**: Total locomotor activity of female/male mice in 23 h, measured in a metabolic cage system (TSE Systems) at week 4 of HFD feeding [n = 10 mice/group, unpaired *t*-test]. **D**: EE of female/male mice normalized to lean body mass, measured at week 4 of HFD feeding, expressed as means ±SEM per hour [n = 10 mice/group, two-way ANOVA with repeated measures] (factor interaction: p_sex/time_<0.001). **E**: Food efficiency (weight gain[g]/[g]food intake) calculated over 15 weeks of HFD-feeding [n = 10 mice/group, two-way ANOVA with repeated measures] (factor interaction: p_sex/time_<0.001). Black columns and symbols represent male mice, white =  female. *p≤0.05, **p≤0.01, # p≤0.001 vs. other sex.

Obesity was induced by feeding a HFD over 15 weeks. Male mice responded to HFD feeding with an accelerated BW-gain compared to female mice. As depicted in Figure 2A BW of male mice was significantly higher compared to females. Accordingly, relative weight increase calculated as percent of initial weight was significantly higher in the male mice (Figure 2B). To evaluate whether this variation in weight gain was caused by differences in metabolic activity mice were phenotyped with regards to locomotor activity, EE and the respiratory exchange ratio (RER) after 4 weeks of HFD feeding. This analysis revealed that females had a significant higher gross locomotor activity (sum of XY-axis movements) (Figure 2C) compared to their male counterparts resulting in higher lean-mass specific EE (Figure 2D). The RER did not differ between the sexes, indicating that substrate utilization was the same in both sexes during initial weight gain (data not shown). To prove the relevance of sexual dimorphic lean-mass specific EE for the observed differences in weight gain we estimated the energy balance for female and male mice (calculated as: energy intake from food – total EE; considering the fecal excretion rate similar between males and females; fecal energy loss was not measured) and determined food efficiency (weight gain/food consumption). As predictable the food consumption was higher in males than in females during HFD-feeding (f 1.85±0.05 g; m 2.32±0.04 g; p<0.001; unpaired *t*-test, mean of 15 weeks HFD) as males had a higher BW and therefore a higher energy demand. The measurement of total EE (not normalized) in the metabolic cage in week 4 of HFD feeding revealed that as expected the total EE was significantly higher in males, as EE increases as a function of BW [20] (f 9.61±0.62 kcal per day; m 11.28±0.79 kcal per day) resulting in a positive energy balance in both sexes (energy balance (total EE [kcal] - energy intake/food [kcal]): f+0.35 kcal per day; m+0.77 kcal per day). This positive energy balance results in weight gain over 15 weeks of HFD-feeding and was slightly higher in male mice; compared to females providing an explanation for accelerated weight gain. However, the calculation of food efficiency showed in the first 9 weeks of HFD-feeding a higher rate of food efficiency in the male mice (Figure 2E). These data corroborate that the stronger response of male mice to HFD is mainly due to lower lean-mass specific EE. We further focused on lean-mass specific EE for an appropriate comparison of female and male mice, since both sexes strongly differ in their BW and amount of lean and fat mass.

### Weight Reduction Phase

After the successful induction of obesity mice were subjected to CR to reduce BW by 20%. Metabolic phenotyping experiments were carried out at two different time points: (1) during the CR-phase representing the dynamic process of BW loss, and (2) after reducing BW by −20% (indicated in the figures as “restriction” and “−20%”, respectively). Mice were sacrificed after reaching the target weight.

**Figure 3:**
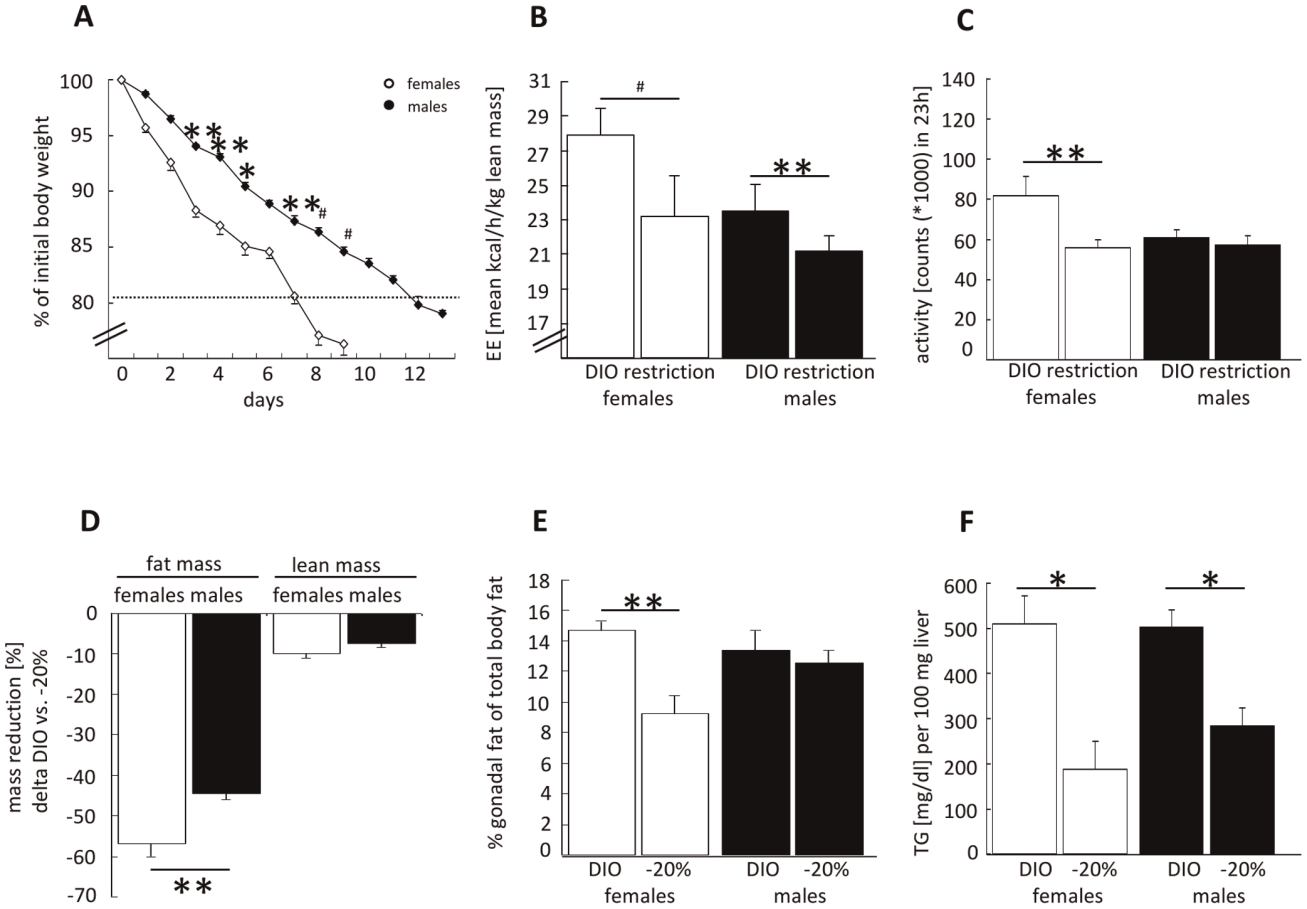
Weight reduction phase. A: Loss of BW in female/male mice during restricted feeding, expressed as percent of DIO-BW. Body weight target: −20% of DIO-BW, for details see method section [n = 10 mice/group, two-way ANOVA with repeated measures] (factor interaction: p_sex/time_<0.001). **B**: Change of lean-mass specific EE measured in female/male mice before weight loss and during caloric restriction. Shown is the mean over 23 h measurement [n = 10 mice/group, two-way ANOVA] (factor interaction: p_sex/weight loss_<0.001). **C**: Total locomotor activity of female/male mice before weight loss and during CR. Shown is total activity during 23 h monitoring [n = 10 mice/group, two-way ANOVA]. **D**: Analysis of body composition (lean and fat mass) in female/male mice calculated as percent reduction (delta: DIO-mass and mass after weight reduction). Lean and fat mass were analyzed separately [n = 10 mice/group, unpaired *t*-test]. **E**: Gonadal fat mass as percent of total fat mass in female/male mice before/after weight reduction [n = 10 mice/group, two-way ANOVA] (factor interaction: p_sex/weight loss_<0.05). **F**: Liver triglycerides measured in female/male mice before/after weight reduction [n = 8 mice/group, two-way ANOVA]. Black and dark grey columns/symbols represent male mice; white and light grey = female mice. DIO = diet-induced obesity or before weight reduction; restriction: during restrictive feeding phase; −20%: after weight reduction, at target weight. *p≤0.05; **p≤0.01; # p≤0.001 DIO vs. −20% or vs. other sex.

The protocol assured an identical caloric reduction (-50%) in both sexes. For better comparison of daily weight loss in female and male mice we calculated the weight reduction as percent of initial weight (Figure 3A). We observed that female mice lost their BW faster than males and reached their target weight within less days compared to male mice (m 12.2 days; f 7.6 days; p<0.001, unpaired *t*-test). The total daily reduction of BW was 0.9±0.2 g in females and 0.9±0.1 g in males which corresponds to an average daily weight loss of 3.3±1.0% and 2.1±0.2% accordingly.

A compensatory reaction to weight reduction is a decrease in EE even higher than would be expected from the decrease in BW. To determine weight-loss-mediated changes in energy metabolism independently from loss in BW and/or lean and fat mass reduction we compared changes in lean-mass specific EE in females and males and energy substrate utilization. These parameters were studied before and during weight loss. As described previously by others [1], [2], both sexes significantly reduced their lean-mass specific EE during weight reduction (Figure 3B), whereas lean-mass specific EE remained generally higher in females. The measurement of locomotor activity (sum of XY-axis movements in 23 h) showed that females responded to BW-reduction with a significant decrease in total activity whereas males did not alter their activity at any time point of measurement (Figure 3C).

Analysis of body composition before and after weight loss revealed that in both sexes reduction of BW was mainly mediated by a loss of fat mass with a significant stronger relative reduction in females than in males (absolute fat mass before and after weight reduction: f fat mass DIO: 9.5±1.3 g; −20%: 4.3±0.9 g; m fat mass DIO: 17.2±0.5 g; −20%: 9.7±0.5 g). In addition, a slight and significant reduction of lean mass could be documented in both sexes but we could not observe any significant difference between male and female mice (Figure 3C, relative reduction of fat and lean mass, delta DIO weight); (absolute lean mass before and after weight reduction: f lean mass DIO: 18.2±0.2 g; −20%: 16.4±0.3 g; m lean mass DIO:24.8±0.5 g; −20%: 22.9±0,4 g). To identify the fat depot responsible for fat mass reduction, gonadal fat pad mass was quantified relative to total fat mass. Females reduced their gonadal fat mass in a disproportionately manner with an augmented loss of gonadal fat relative to total body fat, a process absent in male mice (Figure 3E).

It is well described that under HFD ectopic fat storage e.g. in the liver, skeletal muscle and heart occurs, and is closely related to insulin resistance [21]. Parallel to weight loss these fat stores are reduced, and metabolic alterations improve. As shown in Figure 3F hepatic triglyceride storage was reduced in both sexes.

### Lipolytic Activity

**Figure 4:**
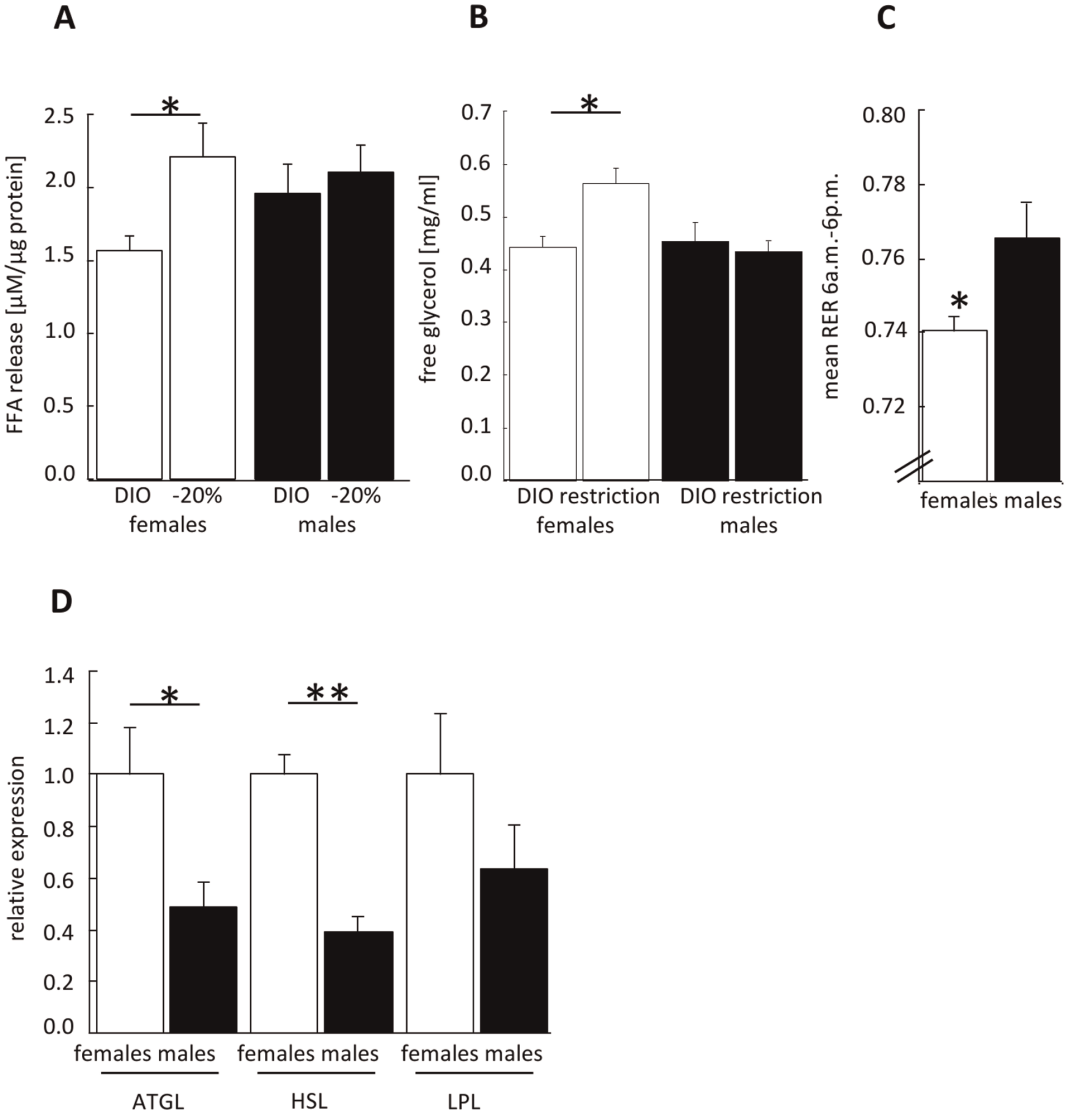
Lipolytic activity. A: Ex-vivo lipolysis assay in murine gonadal adipose tissue explants as a marker of fat-tissue specific lipolytic activity, measured as release of FFA after stimulation with forskolin. Shown is the release of FFA in female/male mice before/after weight reduction [n = 7 mice/group, two-way ANOVA] **B**: Serum concentration of free glycerol in female/male mice before/during weight loss (day 3 of CR) [n = 10 mice/group, two-way ANOVA] ] (factor interaction: p_sex/weight loss_<0.05). **C**: Mean RER during day time (6 a.m.–6 p.m.) in females/males measured during weight loss [n = 10 mice/group, unpaired *t*-test]. **D**: Analysis of ATGL, HSL, and LPL mRNA expression in gonadal-AT from female/male mice after weight reduction. Data are presented as *x*-fold expression of females [n = 8−10 mice/group, unpaired *t*-test, genes were analyzed separately]. Black columns and symbols represent male mice; white: females. DIO: before weight reduction; restriction: during restrictive feeding phase; -20%: after weight reduction, at target weight. *p≤0.05; **p≤0.01 DIO vs. −20% or vs. other sex.

CR resulted in a greater relative reduction of total and gonadal fat mass in female mice compared to male mice suggesting sexual dimorphisms in AT-metabolism. To further investigate sex-specific differences in AT-metabolism, lipolytic rates were assessed in gonadal fat pads before weight reduction (DIO), and after the loss of 20% BW. As outlined in Figure 4A, increased lipolytic capacity during CR was only detectable in AT from female mice. In addition, serum levels of free glycerol, as an indicator of in-vivo lipolysis, was measured. In accordance with the ex-vivo lipolysis assay, serum glycerol exclusively increased in female mice whereas it remained unaffected in male mice (Figure 4B). To evaluate whether these differences in substrate mobilization translate into sexual dimorphic changes in substrate utilization the RER was calculated during CR. At night time (6 p.m.–6 a.m.) a mixed oxidation of carbohydrates, lipids and proteins was measured, equally in both sexes (RER: f 0.84±0.04; m 0.83±0.03; p = 0.75). In contrast, a significant difference between female and male mice was detected during day time as outlined in Figure 4C, suggesting that enhanced lipolytic activity in female mice is associated with a stronger shift in substrate utilization towards lipid oxidation.

To identify underlying mechanisms for the sex-specific responses to CR and regulation of lipolysis we studied mRNA expression of ATGL, HSL, and lipoprotein lipase (LPL) in gonadal fat after weight reduction. ATGL and HSL expression were significantly higher in AT from female mice compared to male mice (calculated relative to the expression level of females). No significant difference between female and male mice could be detected in LPL expression (Figure 4D).

**Figure 5:**
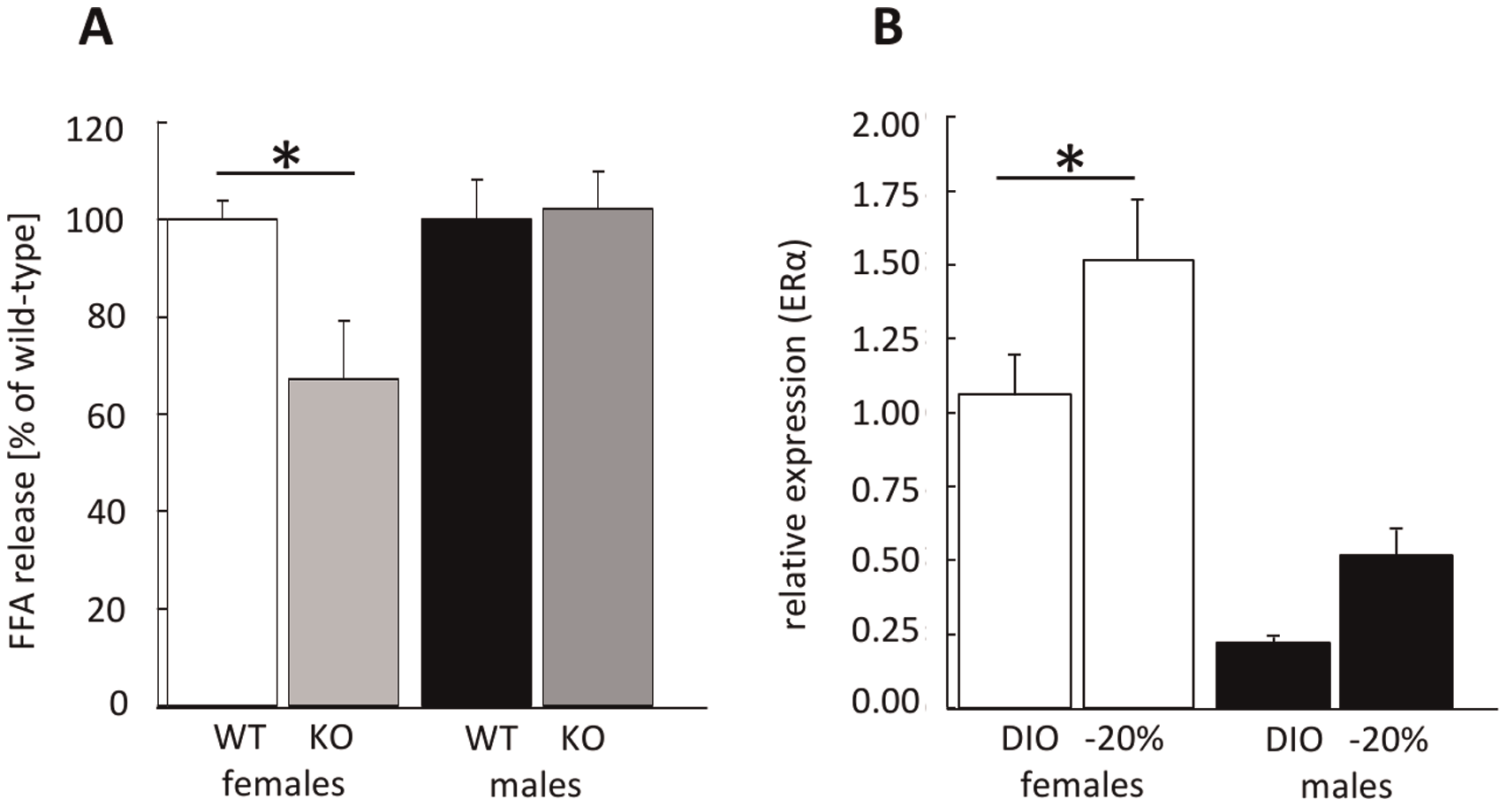
ERα and lipolysis. A: Ex-vivo lipolysis assay in murine gonadal-AT explants from wild-type (WT) and estrogen receptor alpha knock out mice (KO) expressed as percent of WT FFA-release after stimulation with forskolin. Bonferroni posttest showed a significant difference between WT and KO in females [n = 4−5 mice/group, two-way ANOVA]. **B**: Analysis of ERα mRNA expression in gonadal-AT from female/male mice before/after weight reduction. Data are presented as *x*-fold of females (DIO) [n = 9−10 mice/group, two-way ANOVA]. The black/dark grey columns and symbols represent male mice; white/light grey: females. DIO: before weight reduction, –20%: after weight reduction. *p≤0.05 DIO vs. -20% or WT vs. KO.

Estrogen signaling plays a crucial role in weight regulation and lipolytic activity as well as fat distribution. Since we identified sex-specific differences in our model we next tested the importance of estrogen receptors. For this, an ex-vivo lipolysis assay in AT from mice lacking the estrogen receptor alpha (ERαKO) was carried out. Comparing female ERαKO and wild-type animals a significant lower FFA-release was detected in the absence of ERα (Figure 5A). This difference was absent in male mice (Figure 5A). To evaluate whether this observation is related to different expression levels of ERα in female and male mice, we quantified ERα-expression in AT by qPCR before and after weight reduction. As depicted in Figure 5B, CR induced adipose ERα expression in both sexes reaching statistical significance only in females. As expected, female mice had an overall higher expression level of ERα independent of BW suggesting that the sex-dependent impact of ERα on AT-lipolysis results, at least in part, also from the sexual dimorphic expression of the receptor.

### Weight Maintenance and Regain

**Figure 6:**
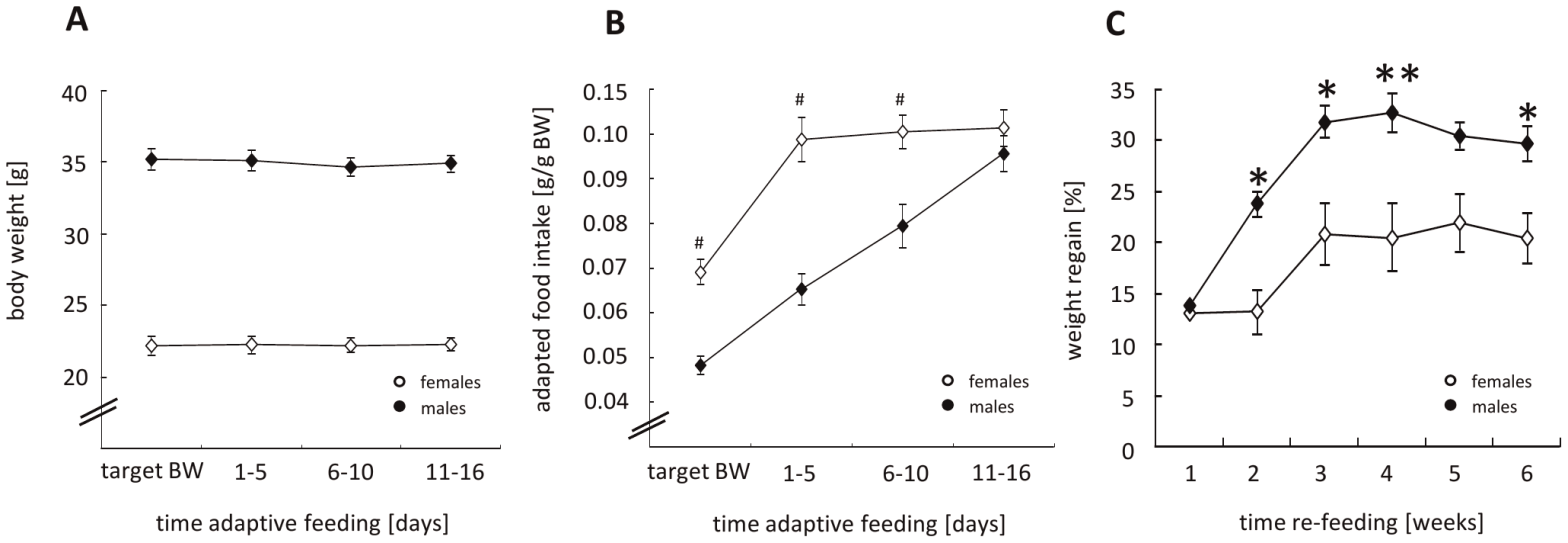
Weight maintenance and regain. A: Stability of body weight of female/male mice during 16 days of adaptive feeding. Shown are the means ±SEM of body weight, measured daily. [n = 10 mice/group] **B**: During adaptive feeding the amount of food was individually adapted to maintain the target weight over 16 days. Shown are the mean amount of given food ±SEM normalized to the BW of female/male mice [n = 10 mice/group, two-way ANOVA with repeated measures] (factor interaction: p_sex/time_<0.001). **C**: Sex-specific differences during weight regain expressed as percent change of body weight before re-feeding. Shown is the BW-development in female and male mice during 6 weeks ad libitum re-feeding. [n = 10 mice/group, two-way ANOVA with repeated measures] (factor interaction: p_sex/time_<0.05). Black symbols represent male mice; white: females. *p≤0.05; **p≤0.01; # p<0.001 vs. other sex.

During the adaptive feeding period of 2 weeks we aimed to maintain the BW as stable as possible (Figure 6A). Therefore, the given food amount was adapted daily to BW-development. Figure 6B shows the corresponding food amount for BW-maintenance in female and male mice. When food intake was normalized to BW, female mice could consume more food than male mice to maintain their BW which may also involve sexual dimorphisms in EE. The food intake in males could be increased continuously reaching the female level at the end of the adaptive feeding period.

To induce weight regain mice were re-fed ad libitum with HFD. At the start of this period, both sexes became hyperphagic and consumed significantly more food than before CR (data not shown). This was similar in male and female mice. Within 6 weeks mice recovered the lost BW, while males regained BW faster than females and in a higher magnitude (Fig. 6C) suggesting that the metabolic sex-differences during weight reduction seem to be preserved in situations of unrestricted energy access.

## Discussion

The present study demonstrates a sex-dependent regulation of weight-gain, -loss, and -regain associated with significant differences in AT lipolytic activity, and substrate utilization/oxidation probably involving ERα-signaling. Under CR female mice reached their target weight faster, predominantly downsized gonadal AT, exhibited an increased rate of ex-vivo lipolysis in gonadal AT associated with significantly increasing glycerol concentrations, lower RER-values, and higher AT expression of the two main lipases ATGL and HSL compared to male animals.

Inititial sexual dimorphisms occurred during the weight gain phase. HFD-induced relative weight gain was significantly higher in male compared to female mice. These data are consistent with previously published results [22], and are likely explained by sexual dimorphic differences in EE. Despite a tendency towards higher (non-normalized) total EE in male mice during weight gain, reversed differences were detected when EE was normalized to lean mass. Female mice exhibited a significant higher EE/g lean mass than males associated with higher locomotor activity and lower food efficiency. In accordance with these results, Xu and colleagues recently demonstrated that deletion of ERα in hypothalamic steroidogenic factor-1 (SF-1) neurons resulted in decreased EE suggesting that sexual dimorphisms during weight gain likely result from central ERα-mediated actions on EE [23]. These authors also demonstrated that floxed-ERα mice crossed with Nestin-Cre transgenic mice resulting in a loss ERα in most brain regions exhibit decreased locomotor activity [23]. In line with our data showing that female locomotor activity during weight gain is higher than male activity, these results support the hypothesis that sex-dependent differences in EE are likely based on sexual dimorphic regulation of locomotor activity in the central nervous system.

During the weight reduction phase sex-specific differences were sustained. Female mice reached their target-BW significantly faster, showed a stronger decrease of total fat mass, and an augmented relative reduction of visceral fat compared to male mice.

To identify the underlying mechanism of these sex-specific differences we firstly investigated energy homeostasis during weight loss. It is well known that under CR humans and rodents reduce their EE for energy conservation, a process responsible for the often observed failure of dieters in maintaining reduced BW [1], [2], [24]. In consonance with previously published reports, female mice had a more pronounced decline in EE than male mice [24]. Despite the stronger decrease female mice still remained on a higher lean-mass specific EE-level than males providing a possible explanation for the sexual dimorphic time dynamics during CR. These data suggest that the sex-dependent CR phenotype is closely linked to the sexual dimorphic phenotype observed during BW gain. However, since EE related sex differences during CR were less pronounced than during BW gain, the presence of additional mechanisms explaining the CR phenotype are likely.

Notably, female mice responded to CR with enhanced reduction of fat mass, in particular of visceral fat mass, when compared to males. Previously, Shi and colleagues demonstrated similar observations in female and male FVBN mice under CR (−60% of daily food consumption). They also showed that female mice predominantly reduced their visceral fat depot in contrast to male mice. In a follow-up study, the authors suggested that regular central leptin signaling via leptin receptors in proopiomelanocortin (POMC) neurons might be involved in sex-specific regulation of fat distribution [25]. Here we identify for the first time sexual dimorphisms in peripheral AT metabolism during CR. Visceral AT-lipolysis during CR vs. the obese DIO state was predominantly induced in female mice whereas this induction was absent in males. This increase in female lipolysis was associated with a significantly higher expression of genes involved in the hydrolysis of triglycerides, ATGL and HSL, when female AT was compared to male AT after CR. ATGL is the rate limiting enzyme involved in AT-lipolysis catalyzing the initial step of triglyceride hydrolysis [26]. Furthermore HSL is a lipase catalyzing the hydrolysis of di-acylglycerol to mono-acylglycerol during the breakdown of triglycerides. Both lipases are important for total breakdown of triglycerides and release of FFAs and glycerol from fat cells. A sex-specific regulation of ATGL has been previously identified by our group were we could show that female mice exhibited higher lipolytic rates during exercise when compared to males [19]. In contrast to the present data, a sexual dimorphic regulation of HSL was not observed in exercise-induced lipolysis suggesting different sex-dependent mechanisms regulating lipolysis during CR and exercise [19]. Future experiments are required to identify sexual dimorphic pathways under varying lipolytic conditions. In summary, our data suggest that both lipases may be involved in the described sex-specific effect on lipolysis during CR.

Together these data could be corroborated in humans demonstrating a greater systemic lipolysis in women during a catecholamine infusion [27]. A number of mechanisms seem to be involved in sex-dependent regulation of AT-lipolysis. The response to different stimuli inducing AT-lipolysis such as catecholamines may vary between the sexes [27]. Here we showed that ATGL and HSL are regulated in a sexual dimorphic manner. A general higher expression level of genes/enzymes involved in ß-oxidation and lipid metabolism in females/women has been described by other groups [18], [28]. We further demonstrated that ERα signaling in AT mediates the female lipolytic phenotype. Since, ERα is also expressed in human AT, and has been identified as an important regulator of adipocyte lipid metabolism [12], [29], one may hypothesize that modulation of ATGL and HSL under the control of ERα translates into sexual dimorphic lipolytic rates. Further studies are required to determine in detail ERα-dependent regulation of AT-lipase expression.

LPL expression and enzyme activity is present in a variety of organs, including AT, heart, skeletal muscle, lung, and others. For most of the organs LPL is regulated in a posttranslational rather than a transcriptional manner, however, a number of *cis*-acting elements in the LPL gene could cause tissue-specific regulation. In AT, the expression of LPL is increased by insulin and food intake and decreased by fasting. LPL-enzymatic activity is dependent on energy requirements and hormonal changes [30] and the main role remains the cleavage of FFA from lipoproteins and their uptake into different tissues. In our study the difference in LPL expression between female and male mice during CR did not reach significance. This was in accordance with our expectations, as LPL expression and activity in AT does not play a major role in times of negative energy balance and lipolysis.

In a model of weight loss, lipolysis is a forced process to reduce fat mass; by triggering a negative energy balance lipolysis is increased to provide FFAs that will be transported from the AT to the skeletal muscle to be used as energy substrate [31]. To investigate these processes in the present study, we measured the RER for analysis of substrate utilization and oxidation. As reviewed from Speakman and Mitchell a key metabolic change in CR is the shift from carbohydrate metabolism to fat metabolism. During the first days of CR mice showed fatty acid oxidation during day time and predominant carbohydrate oxidation after daily feeding [32]. In the present study, higher preference in female mice to use lipids as energy substrate during day time is indicated by significant lower RER-values compared to male mice. This implies that enhanced female lipolysis with increased release of FFAs is paralleled by enhanced rates of systemic FA oxidation.

We mainly focused on sex-specific differences during BW-gain and BW-reduction, however, the present study also demonstrates that sexual dimorphisms continue during the BW-maintenance and –regain phase. During the BW-maintenance phase female mice could consume more food to maintain their BW compared to male mice. Furthermore, female mice exhibited an attenuated BW-response to ad-libitum re-feeding than males during the regain phase. These sex-specific differences are in line with the increased level of female EE compared to males during BW-gain and -loss. In consonance, a recent clinical study in obese/overweight individuals demonstrated a sex-dependent regulation of leptin and ghrelin levels during BW-regain after a hypocaloric diet [33]. Women showed significantly higher levels of leptin during BW-loss and –regain. In addition to its impact on food intake leptin also has profound effects on EE inducing thermogenesis and increasing EE [34]. Thus, sexual dimorphisms in leptin may contribute to sex differences in EE.

The present result may translate into future therapeutic consequences for patients undergoing weight reducing therapy. Increased lipolytic rates in females have been previously described in humans [27]. More importantly, in situations of stimulated lipolysis e.g. exercise or CR, women tend to utilize/oxidize preferentially more lipids than men [35]. In the present study, we could demonstrate that sex-dependent regulation of adipose lipolysis, and FA-oxidation is associated with a faster response to CR in female mice, characterized by a stronger decrease of visceral adiposity, and an attenuation of weight regain. These processes are likely under the control of estrogenic signaling in AT, in particular through ERα. Against the background of an intense research effort on the development of new selective ER-ligands or modulators [36] one may speculate that future pharmacological intervention with tissue-specific, selective ERα-ligands during defined phases of weight reduction (e.g. during early CR or during weight maintenance) may help to support even male dieters in a successful performance of weight loss programs.
